# Supplementary material for: Comparison of body pressure distribution in healthy subjects between bubble wrap and an emergency mattress laid on a cardboard bed: a randomized controlled crossover trial
Source: PeerJ. 2023 Mar 31;11:e15173. doi: 10.7717/peerj.15173 (PMC10069418; doi:10.7717/peerj.15173)
Supplement: Supplemental Information 1 — All data were analyzed using a linear mixed model, and the random effect was assumed as participants nested in sequences adjusted by the variables of age, sex and BMI. Least-squares (LS) means are presented as estimated mean. All 27 subjects lay down on three mattress conditions. A P-value less than 0.05 was considered statistically significant. [file peerj-11-15173-s001.docx]

Analysis results related to Figure 4.

|  | Estimated mean | | | Standard error | | | 95% Confidential Interval | | | F | AIC | BIC | P-value |
| --- | --- | --- | --- | --- | --- | --- | --- | --- | --- | --- | --- | --- | --- |
|  | None | Bubble wrap | Air mattress | None | Bubble wrap | Air mattress | None | Bubble wrap | Air mattress |  |  |  |  |
| Mattress-body contact pressure (mmHg) |  |  |  |  |  |  |  |  |  |  |  |  |  |
| Supine position | 39.68 | 39.29 | 30.18 | 0.80 | 0.60 | 0.60 | 38.04 - 41.32 | 38.05 – 40.54 | 28.95 – 31.40 | 115.25 | 500.11 | 629.89 | < 0.001 |
| Lateral position | 41.17 | 40.20 | 36.33 | 0.76 | 0.52 | 0.72 | 39.60 – 42.74 | 39.14 – 41.26 | 34.83 – 37.82 | 19.68 | 504.64 | 634.47 | < 0.001 |
|  |  |  |  |  |  |  |  |  |  |  |  |  |  |
| Contour area (cm^2^) |  |  |  |  |  |  |  |  |  |  |  |  |  |
| Supine position | 1497.54 | 1547.50 | 2237.56 | 58.07 | 58.07 | 58.07 | 1381.80 - 1613.28 | 1431.76 - 1663.24 | 2121.82 - 2353.30 | 57.02 | 1089.85 | 1096.80 | < 0.001 |
| Lateral position | 1340.82 | 1418.75 | 1971.18 | 45.23 | 45.23 | 45.23 | 1250.71 - 1430.94 | 1328.63 - 1508.87 | 1881.06 - 2061.30 | 60.88 | 1053.09 | 1060.04 | < 0.001 |
|  |  |  |  |  |  |  |  |  |  |  |  |  |  |
| Subjective firmness | 7.62 | 5.75 | 2.07 | 0.26 | 0.31 | 0.21 | 7.09 - 8.16 | 5.11 - 6.39 | 1.63 - 2.51 | 105.37 | 361.51 | 491.29 | < 0.001 |
| Subjective comfort | 6.24 | 4.68 | 2.05 | 0.35 | 0.31 | 0.29 | 5.52- 6.95 | 4.05 – 5.31 | 1.39 – 2.70 | 87.93 | 347.95 | 477.73 | < 0.001 |

Akaike Information Criteria (AIC)

Schwarz's Bayesian Information Criteria (BIC)
