# Supplementary material for: Comparison of body pressure distribution in healthy subjects between bubble wrap and an emergency mattress laid on a cardboard bed: a randomized controlled crossover trial
Source: PeerJ. 2023 Mar 31;11:e15173. doi: 10.7717/peerj.15173 (PMC10069418; doi:10.7717/peerj.15173)
Supplement: Supplemental Information 2 — A Bonferroni correction was performed for the multiple comparisons. All 27 subjects lay down on three mattress conditions. A P-value less than 0.05 was considered statistically significant. [file peerj-11-15173-s002.docx]

Analysis results related to Figure 4.

|  | Mean difference | Standard error | 95% confidential interval | P-value |
| --- | --- | --- | --- | --- |
| **Mattress-body contact pressure (mmHg)** |  |  |  |  |
| **Supine position** |  |  |  |  |
| None vs Bubble wrap | 0.39 | 1.00 | -2.10 – 2.87 | 0.99 |
| None vs Air mattress | 9.50 | 1.00 | 7.03 – 11.97 | < 0.001 |
| Bubble wrap vs Air mattress | 9.12 | 0.85 | 7.01 – 11.22 | < 0.001 |
| **Lateral position** |  |  |  |  |
| None vs Bubble wrap | 0.97 | 0.90 | -1.33 – 3.27 | 0.88 |
| None vs Air mattress | 4.84 | 1.04 | 2.26 – 7.42 | < 0.001 |
| Bubble wrap vs Air mattress | 3.87 | 0.87 | 1.68 – 6.06 | < 0.001 |
|  |  |  |  |  |
| **Contour area (cm^2^)** |  |  |  |  |
| **Supine position** |  |  |  |  |
| None vs Bubble wrap | - 49.96 | 77.21 | -241.23 – 141.32 | 0.99 |
| None vs Air mattress | -740.02 | 77.21 | -931.30 – -548.74 | < 0.001 |
| Bubble wrap vs Air mattress | - 690.01 | 77.21 | -881.34 – 498.78 | < 0.001 |
| **Lateral position** |  |  |  |  |
| None vs Bubble wrap | -77.93 | 62.17 | -231.93 – 76.07 | 0.99 |
| None vs Air mattress | - 630.36 | 62.17 | -784.35 – -476.36 | < 0.001 |
| Bubble wrap vs Air mattress | -552.43 | 62.17 | -706.43 – -398.43 | < 0.001 |
|  |  |  |  |  |
| **Subjective firmness** |  |  |  |  |
| None vs Bubble wrap | 1.87 | 0.39 | 0.89 – 2.86 | < 0.001 |
| None vs Air mattress | 5.55 | 0.32 | 4.76 – 6.35 | < 0.001 |
| Bubble wrap vs Air mattress | 3.68 | 0.39 | 2.78 – 4.58 | < 0.001 |
| **Subjective comfort** |  |  |  |  |
| None vs Bubble wrap | 1.55 | 0.35 | 0.57 – 2.54 | 0.003 |
| None vs Air mattress | 4.19 | 0.33 | 3.36 – 5.03 | < 0.001 |
| Bubble wrap vs Air mattress | 2.64 | 0.28 | 1.93 – 3.35 | < 0.001 |
