# Supplementary material for: Comparison of body pressure distribution in healthy subjects between bubble wrap and an emergency mattress laid on a cardboard bed: a randomized controlled crossover trial
Source: PeerJ. 2023 Mar 31;11:e15173. doi: 10.7717/peerj.15173 (PMC10069418; doi:10.7717/peerj.15173)
Supplement: Supplemental Information 4 [file peerj-11-15173-s004.pdf]

質問紙 / Questionnaire　　　　　　　　　　　　　　　　　　　　　＜Japanese / English＞

調査日　　年　　月　　日 / Date/Month/Year

ID:　.　　性別：　男性　・　女性./ Gender;　Male.　Female　　年齢：　歳　/Age.　　y　　BMI：

BMI ＝ 体重kg ÷ (身長m)²として計算します。

BMI = measured weight (kg) / height² (m)

本研究では以下に該当する方は研究にご参加いただくことができません。該当しないかご確認ください　/Please check if any of them apply to you.

□以下の状況に該当しません/ Not applicable　　□（番号：　）に該当します/Applicable (No.　)

1 :腰背部痛を含む筋骨格系の疼痛または慢性的な痺れを有する方/Person suffering from musculoskeletal or neurogenic pain

2 :仰臥位または側臥位を取ることで疼痛が生じる方/Person with difficulty in supine and lateral positions for pain with positioning

3 :急変の可能性のある疾病を有する方/Person who have a disease that may change suddenly

【記載例】 Example

考えられる中で最大の柔らかさ Most possible softness　0 ——/—— 10　考えられる中で最大の硬さ Most possible firmness

質問１：ご自身が横になられたときに感じた寝心地について伺います/Comfort while lying down

あなたの状態を表す箇所（0-10）に縦線を引いてください/ Please make a cross on the below line (0 -10) that best represents your feelings.

【ダンボールベッド】 Cardboard bed

不快感を全く感じない 最良の寝心地 Most comfortable　0 ———— 10　考えられる中で最悪の寝心地 Worst possible uncomfortable

【気泡緩衝材】 Bubble wrap

不快感を全く感じない 最良の寝心地 Most comfortable　0 ———— 10　考えられる中で最悪の寝心地 Worst possible uncomfortable

【エアマットレス】 Air mattress

不快感を全く感じない 最良の寝心地 Most comfortable　0 ———— 10　考えられる中で最悪の寝心地 Worst possible uncomfortable

質問２：ご自身が横になられたときに感じたベッド（マットレス）の硬さについて伺います/ Firmness while lying down

該当する線分上の箇所に縦線を引いてください/ Please make a cross on the below line (0 -10) that best represents your feelings.

【ダンボールベッド】 Cardboard bed

考えられる中で最大の柔らかさ Most possible softness　0 ———— 10　考えられる中で最大の硬さ Most possible firmness

【気泡緩衝材】 Bubble wrap

考えられる中で最大の柔らかさ Most possible softness　0 ———— 10　考えられる中で最大の硬さ Most possible firmness

【エアマットレス】 Air mattress

考えられる中で最大の柔らかさ Most possible softness　0 ———— 10　考えられる中で最大の硬さ Most possible firmness
