# Supplementary material for: Comparison of body pressure distribution in healthy subjects between bubble wrap and an emergency mattress laid on a cardboard bed: a randomized controlled crossover trial
Source: PeerJ. 2023 Mar 31;11:e15173. doi: 10.7717/peerj.15173 (PMC10069418; doi:10.7717/peerj.15173)
Supplement: Supplemental Information 5 [file peerj-11-15173-s005.docx]

**Study Protocol**

**Study Title**

Comparison of body pressure distribution in healthy subjects between bubble wrap and an emergency mattress laid on a cardboard bed: A randomized controlled crossover trial

**Principal Investigator**

Seiji Hamanishi

**Research Institution**

Nursing Faculty, Kansai University of Social Welfare

**Study Objectives**

This study aimed to investigate the improvement in body pressure distribution and pressure-sensing area when using bubble wrap.

**Study Design**

This trial uses a randomized controlled crossover study. In this trial, all participants receive all the interventions but the order in which they receive the interventions (the sequence) is randomized. All subjects are randomly assigned to two sequences A or B using a random number calculated in Microsoft Excel. This trial is not used by blinding method.

**Inclusion Criteria**

1. People who can give written informed consent (aged between 18 and 60 years).

2. People who can read and fill out explanatory documents, consent forms, and survey forms written in Japanese.

**Exclusion Criteria**

1. People suffering from musculoskeletal or neurogenic pain.

2. People with difficulty in supine and lateral positions for pain with positioning.

3. People who have a disease that may change suddenly.

**Intervention**

A: Cardboard bed without mattress (No mattress)

B: Emergency air mattress

C: Four-folded bubble wrap

**Study Procedures**

**Sequence A**

Cardboard bed without mattress → Four-folded bubble wrap → Emergency air mattress

**Sequence B**

Cardboard bed without mattress → Emergency air mattress → Four-folded bubble wrap

10-minute washout period is inserted between each experimental period.

Body pressure distribution is measured twice for each mattress condition and the average value is calculated.

**Outcome**

**Primary outcome**

Body pressure distribution (the body contact pressure and the body surface contour area)

**Secondary outcome**

VAS (subjective comfort and firmness)

**Other data**

Patient information on individual attributes, including age, sex, and body mass index (BMI).

**Statistical analysis**

A linear mixed-effects model is used to examine the effect of bubble wrap on body pressure distribution. Period, sequence, and mattress were entered into the model as fixed effects, and subjects nested within the sequence are entered as random effects. A Bonferroni analysis is performed to compare the main effects. Age, sex, and BMI were entered into the model as covariates to control for potential confounding factors. In our study, a P-value < 0.05 is considered statistically significant.

**Ethics approval and consent to participate**

This study is performed with the approval of the Ethical review board of Kansai University of Social Welfare. The methods are carried out according to approved guidelines. All study participants provide informed written consent prior to study enrollment. This trial is registered in the University Hospital Medical Information Network Clinical Trials Registry (No.UMIN000048145).
